# Microbial keratinase and the bio-economy: a three-decade meta-analysis of research exploit

**DOI:** 10.1186/s13568-020-01155-8

**Published:** 2021-01-07

**Authors:** Nonso E. Nnolim, Uchechukwu U. Nwodo

**Affiliations:** 1 SAMRC Microbial Water Quality Monitoring Centre, University of Fort Hare, Alice, 5700 South Africa; 2 Applied and Environmental Microbiology Research Group (AEMREG), Department of Biochemistry and Microbiology, University of Fort Hare, Private Bag X1314, Alice, 5700 Eastern Cape South Africa

**Keywords:** Microbial keratinase, Meta-analysis, Collaboration, Sustainable production, Biomass valorization, Biotechnology innovation

## Abstract

Microbial keratinase research has been on an upward trajectory due to the robustness and efficiency of the enzyme toward various green technological processes that promote economic development and environmental sustainability. A compendium of research progression and advancement within the domain was achieved through a bibliometric study to understand the trend of research productivity, scientific impacts, authors' involvement, collaboration networks, and the advancement of knowledge gaps for future research endeavours. A three-decade (1990 to 2019) scholarly published articles were retrieved from the web of science database using a combination of terms "keratinas* or keratinolytic proteas* or keratinolytic enzym*", and subsequently analyzed for bibliometric indicators. A collection of 330 peer-reviewed, research, articles were retrieved for the survey period and authored by 1063 researchers with collaboration index of 3.27. Research productivity was most in 2013 with total research output of 28 articles. The top three authors' keywords were keratinase, keratin and protease with a respective frequency of 188, 26 and 22. India, China and Brazil ranked top in terms of keratinase research outputs and total citation with respective article productivity (total citations) of 85 (1533), 57 (826), and 36 (764). This study evaluated the trend of keratinase research outputs, scientific impact, collaboration networks and biotechnology innovations. It has the potentials to influence positively decision making on future research direction, collaborations and development of products for the bio-economy.

## Key points

- Keratinase has revolutionized proteolytic enzymes’ application potentials.
- The development of keratinase research has been on the upward trajectory.
- This study highlighted the key biotechnology innovations and product developments.

## Introduction

The global reliance of fossil-based resources lingers on, and the long term sustainability of the non-renewable resources remains a mirage (Abejón 2018). The environmental impacts of the products from fossil-based resource have negatively affected the planet earth. Thus, the imperative for a reliable alternative motivates for the exploration of highly abundant renewable resources for sustainable products. The potentials locked in carbonoclastic materials account for the desire to valorize waste biomass into high-value products, and that ensures sustainability in economic development (Akinsemolu 2018; Nnolim et al. 2020a). The agro and food industry generates lots of solid wastes which may suitably feed into the economy with novel valorization approach. Chitinous, lignocellulosic and keratinous biomass are most abundant of the wastes in our environment (Hossain et al. 2007).

Keratinous biomass is typically sturdy due to the high cysteine-mediated disulfide cross-linkages that potentiate structural stability of the polymers, and resilience to attack by biotic and abiotic factors (Schweizer et al. 2006; Wang et al. 2016). The slaughterhouses, leather industries and poultry processing farms are some of the sectors that generate keratinous biomass in large quantities. The need to feed the burgeoning global population has led to increased agroindustrial processes with an inevitable generation of high amounts of wastes. The accumulation of the agro-industrial wastes results in varying degree of pollution in the environs (Kalaikumari et al. 2019).

Environmental protection laws significantly promote efficient management of recalcitrant wastes (Akinsemolu 2018), via recycling to essential products (Stiborova et al. 2016). The microbial valorization of keratinous waste biomass may yield high-quality protein hydrolysates (Mazotto et al. 2017), organic fertilizer (Tamreihao et al. 2017), peptone substituted microbial growth media (Taskin and Kurbanoglu 2011), plant growth hormones (Verma et al. 2016), bioenergy feedstocks (Xia et al. 2012), and industrially important enzymes (Cavello et al. 2018). The physicochemical valorization techniques lack these merits. Consequently, the exploration and exploitation of microbial diversity for novel keratinolytic potentials remains topical (Nnolim et al. 2020b). All the identified microbes associated with keratinolysis are, either bacteria or fungi (Brandelli et al. 2010), and the dermatophytes account for most of the fungal species (Brouta et al. 2001). However, associated virulence traits limit the commercial prospects of the dermatophytes. Therefore, bacteria take precedence as the most viable microbe for keratinous waste bioconversion. Autochthonous and/or allochthonous bacterial strains from different ecological niche have been shown to possess potentials for the bioconversion of keratinous biomass into value-added products; some of the earliest studies started with feather degrading *Bacillus licheniformis* PWD-1 (Williams et al. 1990).

The prospects of keratinases as suitable enzymes for industrial and biotechnological processes include their robustness in withstanding extreme conditions on like the classical proteases (Verma et al. 2017). Microbial keratinases possess unique properties that bestows a potential suitable for use in a green economy including applications in the formulation of functional feed, detergent formulation, bio-decontamination, bating and tanning processes, personal care product formulation, and nanotechnology (Mitsuiki et al. 2006; Paul et al. 2014b; Gupta et al. 2015; Sanghvi et al. 2016; Kalaikumari et al. 2019; Peng et al. 2020). A continuum in the discovery of keratinases with novel properties and function would, significantly, revolutionize the bio-economy landscape, and the prospects presented by proteolytic enzymes. Hitherto, the narrative on microbial keratinases research highlighting milestones such as isolation of producer microbes, optimization of keratinase production, keratin degradation, enzyme purification, biochemical characterization, and the various keratinases application potentials. Regardless of the frequency for which scientific findings are made, and data accumulated on the valorization of keratinous biomass via microbial enzymatic means, there are no explicit documentations delineating research hotspots, lead authors, relevant sources, regional involvement and collaboration networks on the valorization of keratinous biomass via microbial enzymatic means. To develop a comprehensive report from current bibliographic information in the subject area, meta-analytical approach was adopted for the evaluation of the scientific outputs and bio-innovative developments.

Meta-analysis represents an essential method for handling numerous pieces of literature within a field of research (Belter 2015). It a statistical technique aimed at evaluating the significance and trend of research outputs in a localized research field (Cañas-Guerrero et al. 2013). It can be applied systematically to track the progress in a particular research domain. The bibliometric tools prompt the ease of studying subject evolution, comparing regional performance and scientific collaboration within a research niche through bibliometric indicators (Durieux and Gevenois 2010). Bibliometrics has been applied in assessing the research evolution on the impact of lignin valorization from 2000 to 2016 (Abejón et al*.* 2018), hemicellulose valorization from 2000 to 2016 (Abejón 2018), enzyme immobilization from 1991 to 2017 (Gonçalves et al. 2019). The gap identified in the absence of meta-analysis on microbial keratinase research evolution motivates for this study. Therefore, the progression of research on microbial keratinase between 1990 and 2019 was systematically articulated. The meta-analysis was, explicitly, implemented through qualitative and quantitative characterization of the bibliographic information available on the subject matter in the web of science (WoS) core collection database.

## Methods

### Data retrieval

The metadata utilized for the meta-analysis were retrieved from the Clarivate Analytics Web of Science core collection (http://webofknowledge.com/). Web of Science (WoS) is widely used for academic and bibliometric studies as it gives consistent journal coverage of scholarly published articles, high resolution of related records, enhanced metadata for variable information purposes and also, presents more refine options (Li et al. 2018; Birkle et al. 2020). Scientific publications on keratinase studies from 1 January 1990 to 31 December 2019 were retrieved from the WoS on the 10 January 2020 using the key terms "Keratinas* or Keratinolytic proteas* or Keratinolytic enzym*" with title search option. These keywords have been frequently and interchangeably used by authors in the study of microbial keratinases. The incorporation of wildcard (*) and logical operator (or) promoted recovery of relevant published articles with keywords both in singular and plural forms (Capobianco-Uriarte et al. 2019). In addition, title search has been considered most efficient in bibliographic data retrieval against topic search, as it yields more specific results with an infinitesimal loss of sensitivity (Sharma et al. 2018; Olisah et al. 2019). Search results showed a total of 371 publications of different document types. The document types were further refined to exclude review, proceedings, meeting abstract, book chapter, correction, early access and editorial material. After refinement, a collection of 330 articles was retrieved. This document type was chosen as it is generally considered as an original contribution to knowledge (Patience et al. 2017). The documents were further scrutinized for compliance and then, downloaded in the BibTex file format having checked the important fields such as author(s), abstract, addresses, funding information, title, cited references, cited times, language, source, keywords, author identifiers etc. during file exportation. The patented keratinase research discoveries within the three decades of study were retrieved from Google Patents (https://patents.google.com/), by using "Keratinase" as a key term.

### Data analysis

The retrieved data were analyzed for bibliometric indicators using Rstudio software (Rstudio Inc, Boston, USA) with bibliometrix R-package v.3.6.0 (Aria and Cuccurullo 2017). Firstly, the bibliometric analysis was activated in the R environment using the R language "biblioshiny()". The command code opened a biblioshiny web-interface on Google chrome browser which works in synchrony with the R environment. Extracted raw data were imported into the biblioshiny and subsequently converted to a bibliographic data frame. Standard Clarivate Analytics WoS Field Tag codify was used to designate the data frame columns. Descriptive statistics of the dataset implemented include; Author's keyword, keyword plus, annual scientific production, total citation, h_index, most relevant sources, most relevant authors, corresponding author's country, most global cited articles, among others. Bibliographic network matrices were also generated using different bibliographic attributes. Co-occurrence, co-citation, collaboration, among other networks, were automatically computed using bipartite network and visualized by adjusting the network parameters (field, network layout, normalization, node color, clustering algorithm, etc.) and graphical parameters (opacity, number of labels, label size, node shape, edge size etc.) on the biblioshiny web-interface. The percentage frequencies were calculated in Microsoft Excel 2010.

## Results

### The dynamics of article publication on keratinase research

A total of 330 journal articles on keratinase research were retrieved from the WoS during the defined period of survey (1990–2019), which were published by 142 sources. The metadata contained 1063 authors, from which 1058 authors were in multiple authorships with collaboration index of 3.27 (Table 1). Solo authorship was recorded for 6 articles accounting for about 1.8% of the total documents within the study period. The average citation per article on keratinase research during the three decades of survey was 19.74. The articles' publications were distributed among three languages including English (328 articles; 99.4%), Spanish (1 article; 0.6%) and Portuguese (1 article; 0.6%).

**Table 1:** Summary of bibliographic dataset on keratinase researches from 1990 to 2019

Description	Counts
Research articles	330
Sources (journals)	142
Keywords-plus (ID)	635
Author's keywords (DE)	727
Period	1990–2019
Average citations per article	19.74
Authors	1063
Author appearances	1551
Authors of single-authored articles	5
Authors of multi-authored articles	1058
Single-authored article	6
Article per author	0.31
Authors per article	3.22
Co-authors per article	4.70
Collaboration Index	3.27
Language	
English	328
Spanish	1
Portuguese	1

**Fig. 1:**
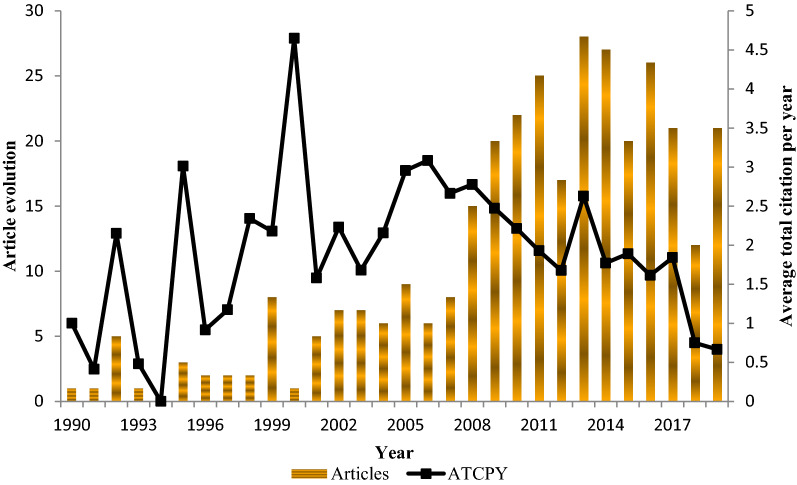
Keratinase research articles published between 1990 and 2019. The article productivity is shown in bar graph; while line graph represents the average total citation per year (ATCPY)

The distribution of articles published on keratinase research between 1990 and 2019 generally showed evolutionary patterns as shown in Fig. 1. Article productivity was relatively low from 1990 to 2000; however, relatively significant number of publications was recorded in 1992 and 1999, with 5 and 8 published articles, respectively. No published article on keratinase research was obtained in 1994 among the retrieved documents. After the year 2000, scientific production increased tremendously with consistent growth in article publication from 2008 to 2013 (Fig. 1). Nonetheless, a sharp decline in the published articles was observed in 2012. The highest number of publications on keratinase research during the survey period was recorded in 2013, with 28 published articles. Beyond 2013, the number of published articles decreased inconsistently. The average total citation per year during the three decades fluctuated across the board, with the maximum citation of 4.65 recorded in 2000.

### Keywords associated with keratinase study: indicators of research hotspots

A collection of 727 author's keywords (DE) and 635 keywords-plus (ID) were associated with the 330 documents retrieved from the WoS (Table 1). Among the twenty most relevant author's keywords, keratinase which is the core focus of the study showed the highest frequency (n = 188), which indicated that about 56.97% of the 330 retrieved articles included keratinase in the itemized author's keywords (Table 2). Other top five ranked author’s keywords included; keratin (n = 26), protease (n = 22), purification (n = 21), and feather degradation (n = 20). The twenty topmost author's keywords could be subdivided into various conceptualizations of which the enzyme's name keratinase, protease, keratinases, and keratinolytic protease form the first clade. The second cluster involves the keratinase inducers (keratin and feather). A common node shoulders the microbial producers (Bacillus, *Bacillus licheniformis*, *Pseudomonas aeruginosa*, *Bacillus pumilus*, and *Bacillus cereus*). Following similar concept, purification, response surface methodology, characterization, optimization, and solid state fermentation are generally anchored on methodology. Furthermore, feather degradation, dehairing and biodegradation represented the potential application of microbial keratinase. Furthermore, 20 most relevant keywords-plus were also presented in Table 2. The topmost five keywords-plus and their frequencies include purification (n = 147), degradation (n = 87), enzymes (n = 67), strain (n = 64) and protease (n = 53). Author's keywords and keywords-plus presented a couple of similar terms, and they are purification, protease, optimization, feather, keratinase, and *B. licheniformis*.

**Table 2:** Top 20 most frequently used keywords on keratinase researches over the survey period

Rank	Author keywords (DE)	Freq. (% of 330)	Rank	Keywords-plus (ID)	Freq. (% of 330)
1	Keratinase	188 (56.97)	1	Purification	147 (44.55)
2	Keratin	26 (7.88)	2	Degradation	87 (26.36)
3	Protease	22 (6.67)	3	Enzymes	67 (20.30)
3	Purification	21 (6.36)	4	Strain	64 (19.39)
4	Feather degradation	20 (6.06)	5	Protease	53 (16.06)
5	Feather	18 (5.46)	6	Alkaline protease	42 (12.73)
6	Bacillus	15 (4.55)	7	Bacterium	40 (12.12)
6	*Bacillus licheniformis*	15 (4.55)	8	Optimization	39 (11.82)
6	Response surface methodology	15 (4.55)	9	Extracellular keratinase	35 (10.61)
7	Dehairing	13 (3.94)	10	Proteinase	35 (10.61)
8	Characterisation	12 (3.64)	11	Feather	34 (10.30)
9	Biodegradation	11 (3.33)	11	Keratinase	32 (9.69)
9	Keratinases	11 (3.33)	12	Keratinolytic serine proteinase	31 (9.39)
9	Optimization	11 (3.33)	13	Expression	30 (9.09)
10	*Bacillus subtilis *	10 (3.03)	13	Enzyme	26 (7.88)
11	*Pseudomonas aeruginosa*	9 (2.73)	14	Subtilis	26 (7.88)
11	Solid state fermentation	9 (2.73)	15	Protein	21 (6.36)
12	*Bacillus pumilus*	8 (2.42)	16	*Bacillus licheniformis*	20 (6.06)
13	*Bacillus cereus*	7 (2.12)	16	Proteins	20 (6.06)
13	Keratinolytic protease^a^	7 (2.12)	16	Keratinolytic activity^b^	19 (5.76)

^a^Thermostability

^b^Serine protease—shared same positions with the keywords in the table superscripted with special characters

### The most impactful keratinase research products: relevance and prospects

The contribution of some articles to the development of keratinase research was assessed by considering the topmost impactful twenty research products in a perking order based on the number of citations they have received during the study period (Table 3). The thrust of the studies may be summarized under the following schemes including keratinse production enhancement, keratinase characterization, keratinous-mediated biomass degradation, keratinase-assisted hide unhairing, keratinase immobilization, keratinase gene cloning and expression and feed nutritional value augmentation with keratinase. The study by Lin and coworkers received the highest number of citation (n = 211) during the three decades of the study, and it was followed by reports of Nam et al. (n = 131), Gradisar et al. (n = 104), Cheng et al. (n = 101), among others (Table 3).

**Table 3:** Twenty most cited journal articles on keratinase research from 1990 to 2019

Rank	Author	Paper title	Journal	Funder^a^	TC		TCPY
1	Lin et al.	Purification and characterization of a keratinase from a feather-degrading *Bacillus licheniformis* strain	Appl Environ Microbiol	NA	211	7.28	
2	Nam et al. (2002)	Native-feather degradation by *Fervidobacterium islandicum* AW-1, a newly isolated keratinase-producing thermophilic anaerobe	Arch Microbiol	NA	131	6.89	
3	Gradisar et al.	Similarities and specificities of fungal keratinolytic proteases: comparison of keratinases of *Paecilomyces marquandii* and *Doratomyces microsporus* to some known proteases	Appl Environ Microbiol	NA	104	6.50	
4	Cheng et al.	Production and characterization of keratinase of a feather-degrading *Bacillus licheniformis* PWD-1	Biosci Biotechnol Biochem		101	3.88	
5	Grazziotin et al. (2006)	Nutritional improvement of feather protein by treatment with microbial keratinase	Anim Feed Sci Technol	NA	94	6.27	
6	Gradisar et al.	Keratinases of *Doratomyces microsporus*	Appl Microbiol Biotechnol	NA	93	4.43	
7	Lin et al.	Nucleotide-sequence and expression of *kerA*, the gene encoding a keratinolytic protease of *Bacillus-licheniformis* PWD-1	Appl Environ Microbiol	NA	89	3.42	
8	Macedo et al.	Novel keratinase from *Bacillus subtilis* s14 exhibiting remarkable dehairing capabilities	Appl Environ Microbiol	NA	87	5.44	
9	Ignatova et al.	Isolation and partial characterization of extracellular keratinase from a wool degrading thermophilic actinomycete *strain Thermoactinomyces candidus*	Can J Microbiol	NA	86	3.91	
10	Mignon et al.	Purification and characterization of a 315 kda keratinolytic subtilisin-like serine protease from *Microsporum canis* and evidence of its secretion in naturally infected cats	Med Mycol	NA	81	3.52	
11	Syed et al.	Production, characterization and application of keratinase from *Streptomyces gulbargensis*	Bioresour Technol	CSIR Task Force Network Programme council of Scientific and Industrial Research (CSIR)—India [NWP 0006]	79	6.58	
12	Konwarh et al.	Polymer-assisted iron oxide magnetic nanoparticle immobilized keratinase	Nanotechnology	The Department of Biotechnology, New Delhi	78	6.50	
13	Tatineni et al.	Purification and characterization of an alkaline keratinase from *Streptomyces* sp.	Bioresour Technol	NA	71	5.46	
14	Pillai and Archana	Hide depilation and feather disintegration studies with keratinolytic serine protease from a novel *Bacillus subtilis* isolate	Appl Microbiol Biotechnol	NA	70	5.38	
15	Wang and Shih	Fermentation production of keratinase from *Bacillus licheniformis* PWD-1 and a recombinant *Bacillus subtilis* fdb-29	J Ind Microbiol Biotechnol	NA	69	3.14	
16	Ramnani and Gupta	Optimization of medium composition for keratinase production on feather by *Bacillus licheniformis* RG1 using statistical methods involving response surface methodology	Biotechnol Appl Biochem	NA	64	3.76	
17	Fakhfakh-Zouari et al.	Application of statistical experimental design for optimization of keratinases production by *Bacillus pumilus* A1 grown on chicken feather and some biochemical properties	Process Biochem	Ministry of Higher Education, Scientific Research and Technology-Tunisia	62	5.64	
17	Suh and Lee	Characterization of a keratinolytic serine protease from *Bacillus subtilis* KS-1	J Protein Chem	NA	62	3.10	
18	Cai et al.	Keratinase production and keratin degradation by a mutant strain of *Bacillus subtilis*	J Zhejiang Univ -Sci B-a	NA	61	4.69	
19	Odetallah et al.	Keratinase in starter diets improves growth of broiler chicks	Poult sci	NA	59	3.28	

*TC* total citation, *TCPY* total citation per year

^*^NA not available

### Keratinase-based products: from laboratory to the market

A few keratinase-based formulations have been made available for commercial uses as presented in Table 4. These products were formulated with keratinases predominantly from *B. licheniformis* strains. PROTEOS Biotech has four branded formulations in the market which include Keratoclean^®^ sensitive PB, Keratoclean^®^ HYDRA PB, Keratoclean^®^ BP and PURE100 KERATINASE. They are specifically used as topical agents for the treatment of skin problems and related conditions. Valkerase^®^ and Versazyme^®^ are products of BioResource International, Inc.; while CIBENZA^®^ DP100 and FEED-0001 were introduced to the feed market by Novus International, Inc. and Creative Enzymes^®^, respectively. These keratinase–based formulations are useful for the improvement of the nutritional values of animal feeds.

**Table 4:** Keratinase–based formulation in commercial use (some information were adapted from Hassan et al. 2020 and Srivastava et al. 2020)

Enzyme	Microbial producer	Formulation	Uses^a^	Manufacturer/supplier
Keratinase	*B. licheniformis*	Keratoclean^®^ sensitive PB	Skin care products	PROTEOS Biotech
Keratinase	*B. licheniformis*	Versazyme^®^	Animal feed preparation, keratin and collagen degradation	BioResource International, Inc
Keratinase	*B. licheniformis*	Valkerase^®^	Feather meal processing	BioResource International, Inc
Keratinolytic protease	*B. licheniformis*	CIBENZA^®^ DP100	Poultry and swine feed ingredient digestion	Novus International, Inc
Keratinase	*B. licheniformis*	Keratoclean^®^ HYDRA PB	Skin care products	PROTEOS Biotech
Keratinase	*B. licheniformis*	Keratoclean^®^ BP	Skin care products	PROTEOS Biotech
Keratinase	*B. licheniformis*	Prionzyme M	Biodecontamination	Genencor International, Inc
Keratinase	*B. licheniformis*	FEED-0001	Insoluble keratin degradation	Creative Enzymes^®^
Keratinase	Recombinant *Escherichia coli* BL21	NATE-0853	Enzymatic treatment of EB, GAGs and cells	Creative Enzymes^®^
Keratinase	*B. licheniformis*	PURE100 KERATINASE	Biomedical, pharmaceutical and cosmetic applications	PROTEOS Biotech

^a^*EB* elementary body, *GAGs* glycosaminoglycans

### Keratinase research innovations and patents

A number of innovative discoveries, pertaining keratinase research, have been patented over the past three decades, and this signified a milestone in this field of research. The bio-innovations may be placed under the following broad categories but not limited to process design, method development and product formulation. The title of the invention, patent number, region, inventor and legal status of the patent are presented in Table 5. A patent grants the inventor an exclusive right of sole ownership of a product or process for a defined period that the invention is protected. Some keratinase invention patents are functionally active. While others have variable legal status, such as expired, abandoned, discontinued, terminated, pending, withdrawn, and invalid (Table 5). The reasons advanced for the various aforementioned status included patent expiration, post-publication rejection of the patent application, application awaiting examination and approval and intellectual property (IP) right cessation due to unpaid dues. Among the regions that the inventors are domiciled, China recorded the highest number of keratinase patents which invariably indicated the participation of prolific researchers from this region.

**Table 5:** A few selected keratinase research innovation patents in the past three decades (1990–2019)

Title	Patent number	Region	Weblink	Inventor	Legal status^a^
Immobilization of keratinase for proteolysis and keratinolysis	US20030108991A1	United States	https://patents.google.com/patent/US20030108991A1/en	Shih et al.	Abandoned
Keratinase mutant that a kind of substrate specificity improves and preparation method thereof	CN104726436B	China	https://patents.google.com/patent/CN104726436B/en	Zhang et al.	Active
Keratinase and production method thereof	JP5699261B2	Japan	https://patents.google.com/patent/JP5699261B2/en	Watanabe et al.	Active
Novel metal ion-tolerant keratinase and application thereof	CN108060170B	China	https://patents.google.com/patent/CN108060170B/en	Shi et al.	Active
Keratinase mutant with improved thermal stability and preparation method thereof	CN104017791B	China	https://patents.google.com/patent/CN104017791B/en	Chen et al.	Active
Development of an asporogenic *Bacillus licheniformis* and production of keratinase therefrom	US20060127982A1	United States	https://patents.google.com/patent/US20060127982A1/en	Jason Shih	Abandoned
Hari cosmetic composition comprising keratinase for protection of hair and repair of hair damage	KR101207490B1	South Korea	https://patents.google.com/patent/KR101207490B1/en	Kang and Kim	Terminated
Alkali keratinase, dna encoding the same and method for using the same	JP2011155932A	Japan	https://patents.google.com/patent/JP2011155932A/en	Inoue et al.	Pending
Keratinase and gene sequence and application method thereof	CN106350530A	China	https://patents.google.com/patent/CN106350530A/en	Gong et al.	Active
Signal peptide for optimizing efficient secretory expression of keratinase (Ker) and application thereof	CN107200772B	China	https://patents.google.com/patent/CN107200772B/en	Zeng et al.	Active
A kind of fermentation process efficiently producing keratinase using recombination bacillus coli	CN105316306B	China	https://patents.google.com/patent/CN105316306B/en	Zhang et al.	Active
A kind of isolation and purification method of keratinase and application	CN105821023B	China	https://patents.google.com/patent/CN105821023B/en	Bian et al.	Active
Keratinase mutant that a kind of catalytic rate improves and preparation method thereof	CN105002152B	China	https://patents.google.com/patent/CN105002152B/en	Zhang et al.	Active
Cleaning agent containing keratinase	DE102016214383A1	Germany	https://patents.google.com/patent/DE102016214383A1/en	Walter Haberlein	Withdrawn
Keratinase produced by *Bacillus licheniformis*	US5877000A	United States	https://patents.google.com/patent/US5877000A/en	Burtt and Ichida	Expired
Detergent containing keratinase	DE102016214382A1	Germany	https://patents.google.com/patent/DE102016214382A1/en	Haberlein and Stehr	Withdrawn
Enzymatic formulation of lotion containing microbial keratinase as sole depilatory agent and drug absorption promoter	BR102014021563A2	Brazil	https://patents.google.com/patent/BR102014021563A2/en	Macedo et al.	Discontinued
Heat stable keratinase and use thereof	DK3027746T3	Denmark	https://patents.google.com/patent/DK3027746T3/en	Ho et al.	Active
A method of preparing feather albumen powder using alkali protease and keratinase	CN104798981B	China	https://patents.google.com/patent/CN104798981B/en	Hao et al.	Terminated
It is a kind of produce keratinase *Pseudomonas aeruginosa* and its application	CN105861373B	China	https://patents.google.com/patent/CN105861373B/en	Huang et al.	Active
Method of preparing feather protein powder from keratinase	CN104770574A	China	https://patents.google.com/patent/CN104770574A/en	Lujiang et al.	Terminated
Pearl albefaction method mediated by keratinase and combined with redox	CN100579412C	China	https://patents.google.com/patent/CN100579412C/en	Zhang et al.	Active
Produce *Bacillus cereus* and its application of keratinase	CN107868762A	China	https://patents.google.com/patent/CN107868762A/en	Bian et al.	Active
Method for producing amino acid by hydrolyzing feather with keratinase	CN110760550A	China	https://patents.google.com/patent/CN110760550A/en	Zhang et al.	Active
Strain of bacterium *Bacillus licheniformis* as producer of keratinase	RU2177994C2	Russia	https://patents.google.com/patent/RU2177994C2/en	Tsurikova et al.	Invalid
A kind of compounding of keratinase and application in the industrial production	CN107916308B	China	https://patents.google.com/patent/CN107916308B/en	Shi et al.	Active
Novel keratinase derived from thermophilic bacteria and use thereof	KR101785613B1	South Korea	https://patents.google.com/patent/KR101785613B1/en	Lee et al.	Active
Heat stable keratinase and uses thereof	TWI564390B	Taiwan	https://patents.google.com/patent/TWI564390B/en	Mengqiao et al.	N/A
Method for producing bacterial strain and efficiently degrading feathers by using keratinase	CN107828847B	China	https://patents.google.com/patent/CN107828847B/en	Zhang et al.	Active
DNA encoding *Bacillus licheniformis* PWD-1 keratinase	US5712147A	United States	https://patents.google.com/patent/US5712147A/en	Shih et al.	Expired
The keratinase mutant and application that a kind of catalytic performance improves	CN109593746A	China	https://patents.google.com/patent/CN109593746A/en	Shi et al.	Active
The method and its application of gold nanoparticle preparation are carried out using keratinase	CN109456958A	China	https://patents.google.com/patent/CN109456958A/en	Yu et al.	Active
A kind of keratinase mutant being transformed through thermal stability	CN108949729A	China	https://patents.google.com/patent/CN108949729A/en	Gong et al.	Active
*Stenotrophomonas maltophilia* for generating keratinase and application of *Stenotrophomonas maltophilia*	CN102329751B	China	https://patents.google.com/patent/CN102329751B/en	Chen et al.	Active
Removal of biological deposits	US20200315943A1	United States	https://patents.google.com/patent/US20200315943A1/en	Speight and Navone	Pending
Cosmetics for hair removal	JP3923424B2	Japan	https://patents.google.com/patent/JP3923424B2/en	Maeda and Yamamoto	Active
Composition and method for destruction of infectious prion proteins	US6613505B2	United States	https://patents.google.com/patent/US6613505B2/en	Jason Shih	Active
Degradation of beta-amyloid proteins with keratinase	US20170042980A1	United States	https://patents.google.com/patent/US20170042980A1/en	Kirk Johnson	Abandoned
A method of improving keratinase vigor	CN109797145A	China	https://patents.google.com/patent/CN109797145A/en	Wang et al.	Active

^a^N/A not available

### Keratinase research evolution by country

The countries' productivity was ranked based on the total number of research articles emanating from the various countries where the corresponding authors are domiciled. Among the top twenty most productive countries on keratinase research, India ranked first with 85 publications, distributed into single country (78 articles) and multi-countries (7 articles) productions, representing 25.99% of the total research outputs published between 1990 and 2019. These publications have been cited 1533 times, with an average article citation of 18.04 (Table 6). In the same vein, the People's Republic of China and Brazil were ranked second and third countries, with 57 (17.43%) and 36 (11.01%) articles, respectively. The total citations recorded for the both countries were 826 for China and 764 for Brazil. South Korea and the USA occupied same position with 13 (3.98%) publications from each region. However, total citations of the two countries differed significantly; 326 for the USA and 311 for South Korea. Egypt was ranked fifth with 12 (3.67%) articles. Other African countries on the list include Algeria, Tunisia and Nigeria. Algeria and Tunisia occupied tenth position with 5 articles each, while Nigeria ranked eleventh, with 4 published articles. Hungary shares twelfth position with Malaysia and Turkey, each contributing 3 papers during the survey period.

**Table 6:** The top twenty productive countries on keratinase research based on corresponding authors affiliation

CP_BCA_						TC_PC_			
Rank	Country	Articles	Freq. (%)	SCP	MCP	Rank	Country	TC	AAC
1	India	85	25.994	78	7	1	India	1533	18.04
2	China	57	17.431	51	6	2	China	826	14.49
3	Brazil	36	11.009	35	1	3	Brazil	764	21.22
4	South Korea	13	3.976	7	6	4	USA	326	25.08
4	USA	13	3.976	12	1	5	South Korea	311	23.92
5	Egypt	12	3.67	9	3	6	Slovenia	298	59.60
6	Japan	11	3.364	11	0	7	Japan	296	26.91
7	Taiwan	10	3.058	9	1	8	Tunisia	215	43.00
8	Argentina	9	2.752	7	2	9	Taiwan	195	19.50
9	Belgium	6	1.835	3	3	10	Belgium	187	31.17
9	Iran	6	1.835	6	0	11	Germany	175	35.00
9	United Kingdom	6	1.835	4	2	12	Egypt	150	12.50
10	Algeria	5	1.529	1	4	13	United Kingdom	143	23.83
10	Germany	5	1.529	3	2	14	Venezuela	91	45.50
10	Poland	5	1.529	5	0	15	Argentina	82	9.11
10	Slovenia	5	1.529	4	1	16	Nigeria	80	20.00
10	Tunisia	5	1.529	2	3	17	Algeria	64	12.80
11	Nigeria	4	1.223	2	2	18	Czech Republic	63	21.00
12	Czech Republic	3	0.917	1	2	19	Hungary	55	18.33
12	Hungary^a^	3	0.917	2	1	20	France	45	22.50

*CP*_*BCA*_ country productivity based on corresponding author, *SCP* single country production, *MCP* multiple countries production, *TC* total citations, *AAC* average article citations, *TC*_*PC*_ total citation per country

^a^Malaysia and Turkey also ranked twelfth position

### Collaboration networks: efficient tools for the improvement of knowledge base

**Fig. 2:**
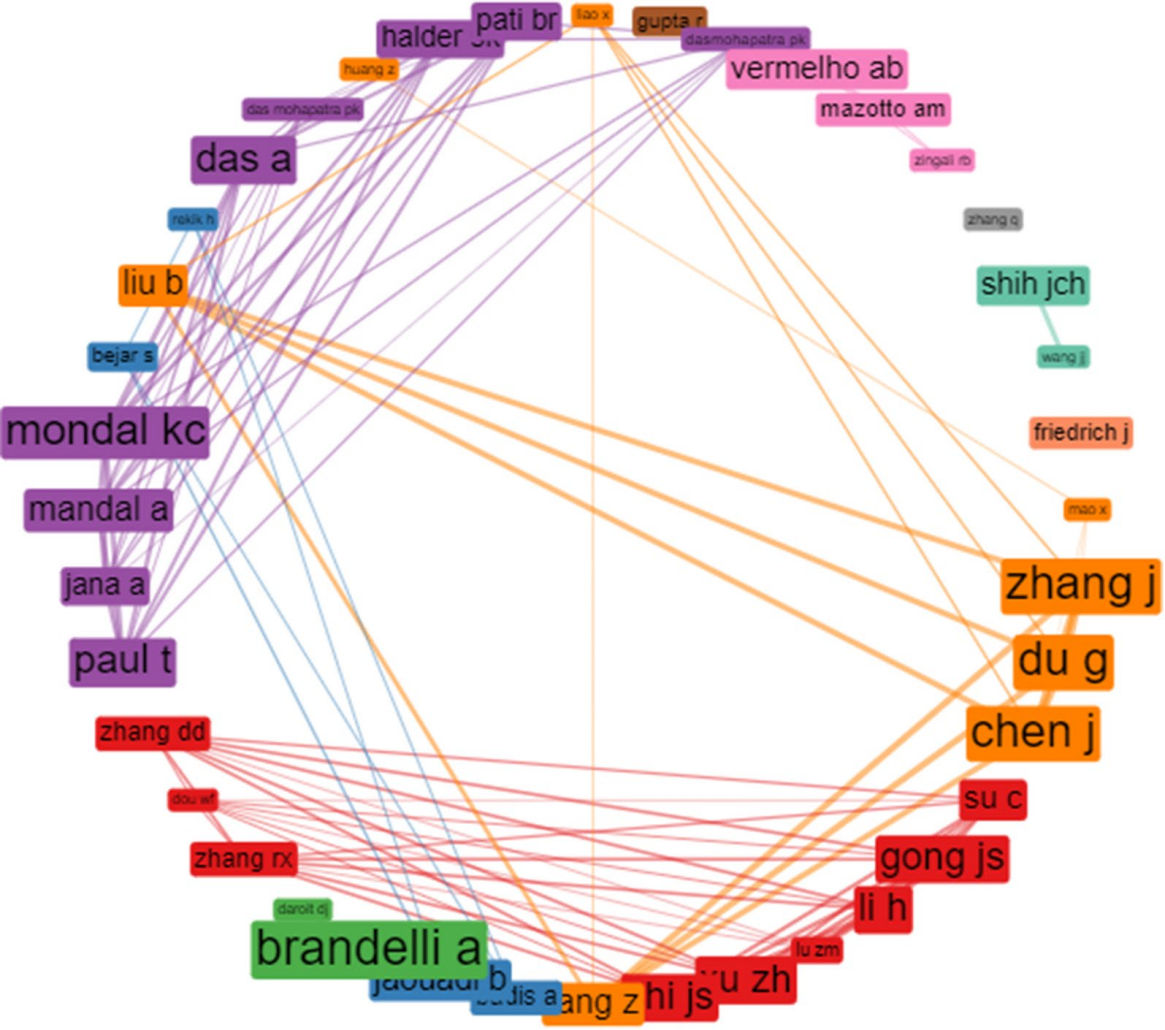
The top forty authors' collaboration network on keratinase research. Each node represents a single author, while the collaboration pathways are the connecting lines. The thickness of the lines indicates the strength of the collaboration

Figure 2 showed the collaboration network of the top 40 authors on keratinase research. The network displayed unique collaboration of authors from the same region/country. For instance, the authors’ collaboration displayed in yellow network that included Fang Z, Liu B, Zhang J, Du G, Cheng J, and others involved authors from China. Similarly, Zhang DD, Zhang RX, Gong JS, Su C and the other authors on the red network are also domiciled in China. In the same vein, Brandelli A. and Daroit DJ that appeared in green network hailed from Brazil. The purple coloured collaboration network that involved Paul T, Mondal KC and collaborators constituted authors from India. Shih JCH and Wang J from the USA are pioneers in the keratinase research field. Similar pattern of regional collaboration is applicable to other authors that share similar network connection. The authors without visible collaboration network could be that they are not collaborating with any authors on the top forty or they are independent researchers. Notably, among the lead authors of keratinase research, Brandelli A and Shih JCH recorded the highest number of published articles (18) and total citations (656) respectively (Additional file 1: Table S1).

**Fig. 3:**
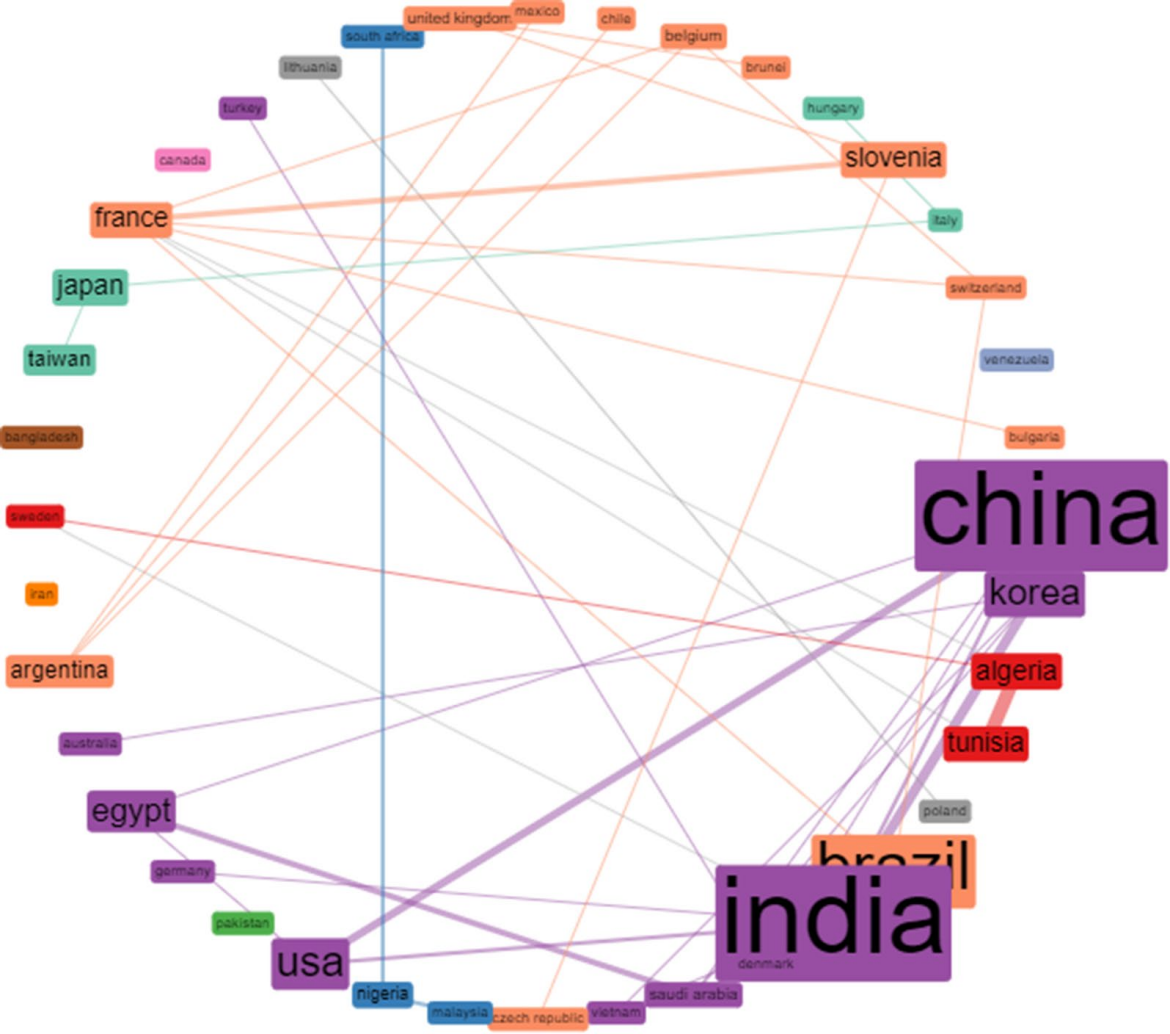
Collaboration network of 40 top listed countries on keratinase research. The size of the node indicates the frequency of collaboration within countries, while the thickness of the network shows the strength of collaboration between countries

The collaboration networks of top 40 countries are shown in Fig. 3. The collaboration networks among various countries are presented in different colours. Larger nodes demonstrate greater number of collaborations within countries. The thickness of network linkages showcases the strength of collaboration between the two countries. China formed strong collaboration network with India and the USA, while collaborating in varying degrees with Saudi Arabia, Vietnam and Egypt. France formed cluster with Slovenia, Belgium, Switzerland, Bulgaria, Algeria, Tunisia and Brazil. Similarly, Nigeria has South Africa and Malaysia in its network domain. Conversely, Venezuela, Canada, Bangladesh, Iran and Pakistan did not form collaborative clusters with any topmost forty countries that have published scholarly articles on keratinase research during the three decades of study.

## Discussion

The evaluation of the macro-state of keratinase research, based on the information available from the WoS database, generally showed a trajectory growth pattern of research products. Notably, the recent past decade recorded the highest number of publications and this may be attributed to the involvement of more active researchers, adequate research funding, availability of state-of-the-art facilities and most importantly, the foreseeable application prospects of microbial keratinase in green technology (Rosenbloom et al. 2015; Hassan et al. 2020).

Keywords associated with keratinase research spotlight the hotspots and dimensions of researches over the study period. Author keywords are useful in the identification of scientific concepts advanced by an article. Their frequency therefore indicates the authors’ involvement, and also expresses the trend of research in that particular field (Tripathi et al. 2018). Keywords-plus which the WoS extracts automatically from the metadata of a particular research field as indexing terms also assist in the determination of a knowledge structure, although they may be less comprehensive in divulging the intrinsic aim of a research (Zhang et al. 2016). It may be inferred from the keywords frequency that bacterial keratinases have been predominantly explored, of which the lead producers are *Bacillus* spp. (Nnolim et al. 2020b). Animal hide dehairing potentials of some candidate keratinases from various bacterial strains have been adequately evaluated which may serve as green depilatory strategy in leather processing (Fang et al. 2017a). However, there are yet to be commercially marketed keratinase based products for the processing of hides and skins in leather production. The cost-efficient and eco-friendly nature of these bio-based processes have become an attraction for biotechnologists, hence the continuum in the development of the field. Moreso, a few empirical experimentations have lately suggested other promising applications of keratinase which include nanotechnology, bio-bleaching, and bio-energy (Gupta et al. 2015; Patinvoh et al. 2016; Zhang et al. 2019). Keratinases from wild microorganisms have been predominantly characterized and evaluated for various biotechnological and industrial advancements (Srivastava et al. 2020). However, a couple of limiting factors including prolonged fermentation period and low enzyme yield may thwart the commercial prospects of these keratinases. Therefore, cloning and overexpression vis-à-vis molecular optimization of the keratinase expression in industrial suitable hosts portend the tendency to ameliorate the productivity hitch fostered by the physiology of the wild microbial producers (Fang et al. 2019).

The most cited articles were published during the first two decades of the survey period and the significant number of citations accrued by these papers may suggest their relevance and scientific contributions toward the development of keratinase research field (Agarwal et al. 2016). Notably, with an exception of three studies, the topmost cited papers were basically self-funded research. This might have contributed to the fluctuations in the number of research outputs and sluggish development of keratinase research during the first decades of the survey period. Research funding has been identified as a catalyst that promotes and encourages scientific development and innovation (Rosenbloom et al. 2015). Therefore, it is imperative that funding agencies and other stake holders adequately support this research niche that intrinsically drives bio-based industry for sustainable bioeconomy.

The study by Lin et al. was an advancement of the work previously reported by Williams et al. (1990) on feather degradation potential of *B. licheniformis* PWD-1 isolated from a laboratory poultry waste digestor. The discovery of this bacterial isolate landscaped new vistas of exploiting microbial keratinases as important candidates in green technology. Hence, the exponential growth and development of keratinase research in the recent past were based on the fundamentals of strain PWD-1characterization and its offshoots including PWD-1 keratinase characterization (Cheng et al. 1995), PWD-1 keratinase gene (*kerA*) cloning (Lin et al. 1995), PWD-1 keratinase gene expression in *B. subtilis* (Lin et al. 1997), comparative keratinase activity of *B. licheniformis* PWD-1 and recombinant *B. subtilis* FDB-29 (Wang and Shih 1999), PWD-1 keratinase immobilization (Wang et al. 2003).

The robust keratinolytic system of *B. licheniformis* therefore prompted the development of a few keratinase based formulations at commercial scale. Therefore, BioResource International, Inc. formulations; Versazyme^®^ and Valkerase^®^ preparations were efficient and sustainable means of utilizing avian feather wastes as low cost and good quality protein sources for animal husbandry (Potera 2013). Evaluation of Versazyme on the growth performance of birds showed that it significantly improved the feed conversion, body weight, and breast yield of poultry birds (Wang et al. 2006). The keratinase inclusion in animal feeds promotes the bioavailability of essential proteins which ultimately improves nutrient utilization and less excretion of nitrogen by fed animals (Potera 2013). Furthermore, the products of PROTEOS Biotech were formulated by microencapsulation of keratinase from *B. licheniformis.* These gentle natural formulations serve as alternatives to the synthetic alpha hydroxyl acids (AHAs), urea and thioglycolates predominantly utilized in cosmetics as cell renewing, moisturizing, and anti-hair growth agents. The commercialization of keratinase–based formulations presents the prospects of sustainably revolutionizing various sectors of the bioeconomy, while at the same time mitigating environmental pollution that could have been potentiated by conventional chemicals.

India performance in terms of keratinase research suggests the involvement of many active researchers from this region. The significant number of articles recorded by a few countries may be attributed to the availability of requisite facilities, adequate research funding, and good collaboration network (Lee and Bozeman 2005; Rosenbloom et al. 2015). Generally, the countries on the list were more in intra-national publication than multi-national production and this single country production considerably promoted scientific productivity (Scarazzati and Wang 2019).

The researchers, in their respective collaboration networks, have contributed significantly to the development of keratinase research and innovation. Paul and collaborators have sufficiently advanced the use of microbial keratinase for different benefits including valorization of keratinous wastes into high value functional peptides and essential amino acids (Paul et al. 2014a, b), cleaning properties of keratinase with an admixture of detergent (Paul et al. 2014a, b), plant growth promotion by keratinase-derived organic hydrolysates (Paul et al. 2013) and many other innovative studies.

Fang and coworkers have shown their expertise by the discovery of novel keratinases from *Stenotrophomas maltophilia* (Fang et al. 2013a), keratinase production process optimum construction (Fang et al. 2013b), the biotechnological development of the bacterial strain for the overproduction of keratinase (Fang et al. 2014). They have also employed various protein engineering approaches to improve keratinase biotechnological and industrial values which were detailed in studies including truncation of keratinase PPC domain for catalytic efficiency improvement (Fang et al. 2016a), keratinase domain exchange for an improved catalytic efficiency (Fang et al. 2016b), keratinase substrate specificity alteration (Fang et al. 2015), thermostabiliy improvement through rational protein engineering approaches (Fang et al. 2017b), and cloning and overexpression of keratinase in an heterologous industrial host (Fang et al. 2019). These rigorous investigations therefore underpin the industrial and biotechnological potentials of *S. maltophilia* keratinolytic protease.

PWD-1 keratinase was a product of Shih, Wang and collaborators’ innovative researches (Wang and Shih 1999; Wang et al. 2004). Its dexterity in degradation of recalcitrant keratinous wastes and augmentation of their nutritional value has become an attraction for researchers, nutritionists and feed producers. Consequently, the formulation of two patented products Versazyme^®^ (launched 2005) and Valkerase^®^ (launched 2006) by Shih and coworkers at BioResource International was on the basis of this thermostable keratinase (Potera 2013). The Shih founded BioResource International further extended the market of keratinase-based products outside the US in 2008 by partnering with a China based animal nutritional company Novus International. This partnership promoted the distribution of these keratinase-based formulations in the Asian market which attracted significant revenues (Potera 2013). Therefore, scientific collaboration of researchers in a particular research domain ensures expansion of knowledge base and promotes sustainable growth and development of that field (Fang et al. 2020). Funding bodies especially government agencies strictly facilitate collaboration as one of the formal contractual agreements during grant awards (Lee and Bozeman 2005), as it brings about cross-pollination of ideas that would enhance productivity and innovation. International collaboration of scientific community fosters high quality scientific production and also promotes mobility of researchers with the ultimate goal of enhancing their scientific capacity (Chinchilla-Rodríguez et al. 2018). Moreso, networking of researchers at both domestic and international levels positively influences the growth of budding researchers by provision of new insights and career boost (Scarazzati and Wang 2019). However, international collaborations could suffer variable hiccups but not limited to language barrier, project delay, travel issues and political instability which may affect research productivity and innovation.

In conclusion, this study provided insight on the development of keratinase research domain vis-à-vis research outputs, scientific impacts, authors' involvement, collaboration pathways and bioinnovations over the past three decades. This study would imperatively subsidize the time investment by researchers to understand the trends of researches, fundamental hotspots, as well as assisting in the identification of knowledge gaps and prioritization of future research endeavours for innovative developments. The commercially available keratinase-based products currently in use are either for bio-decontamination, upgrading of animal feed nutritional value or skin care. Therefore, there is a need to carefully develop commercial keratinase-based products that lack collagenase activity for sustainable production of high quality leather materials. Moreso, other bacterial keratinases that have shown promising properties may be exploited for the development of novel products at commercial scale since all the existing keratinase-based products were based on *B. licheniformis* keratinases.

## Supplementary Information

**Additional file 1: Table S1**. Top 20 most prolific authors of keratinase research articles between 1990 and 2019.

## Data Availability

The datasets analyzed in the present study are available from the corresponding author on reasonable request.
