# Supplementary material for: Microbial keratinase and the bio-economy: a three-decade meta-analysis of research exploit
Source: AMB Express. 2021 Jan 7;11:12. doi: 10.1186/s13568-020-01155-8 (PMC7790984; doi:10.1186/s13568-020-01155-8)
Supplement: Supplementary file 1 — Additional file 1: Table S1. Top 20 most prolific authors of keratinase research articles between 1990 and 2019. [file 13568_2020_1155_MOESM1_ESM.pdf]

AMB Express

Microbial keratinase and the bio-economy: A three-decade meta-analysis of research exploit

Nonso E. Nnolim[1,2] and Uchechukwu U. Nwodo[1,2,*]

[1]SAMRC Microbial Water Quality Monitoring Centre, University of Fort Hare, Alice, 5700, South Africa

[2]Applied and Environmental Microbiology Research Group (AEMREG), Department of Biochemistry and Microbiology, University of Fort Hare, Private Bag X1314, Alice, 5700 Eastern Cape, South Africa

*Corresponding author; email: UNwodo@ufh.ac.za; tel.: +27406022693; ifax: +27862707453

Table S1 Top 20 most prolific authors of keratinase research articles between 1990 and 2019

Rank	Author	Affiliation	Country	Article	h_index	TC	PY_start
1	Brandelli A	Universidade Federal do Rio Grande do Sul	Brazil	18	12	449	2003
2	Chen J	Jiangnan University	China	13	9	243	2008
2	Du G	Jiangnan University	China	13	9	206	2013
2	Gupta R	University of Delhi	India	13	9	261	2004
2	Shih JCH	North Carolina State University	USA	13	12	656	1992
2	Zhang J	Jiangnan University	China	13	9	206	2013
3	Fang Z	Jiangnan University	China	10	9	161	2013
3	Friedrich J	National Institute of Chemistry	Slovenia	10	9	424	1999
4	Lin H	National Taiwan Ocean University	Taiwan	9	4	42	2015
4	Liu B	Jiangnan University	China	9	8	183	2015
4	Mondal KC	Vidyasagar University	India	9	7	124	2012
5	Das A	Vidyasagar University	India	8	6	112	2012
5	Paul T	Vidyasagar University	India	8	6	97	2014
5	Wang JJ	North Carolina State University	USA	8	7	248	1999
6	Daroit DJ	Universidade Federal do Rio Grande do Sul	Brazil	7	6	150	2010
6	Gong JS	Jiangnan University	China	7	4	42	2015
6	Mandal A	Raja Narendra Lal Khan Women's College	India	7	6	96	2014
6	Sharma R	University of Delhi	India	7	5	68	2010
6	Shi JS	Jiangnan University	China	7	4	42	2015
6	*Vermelho AB	Universidade Federal do Rio de Janeiro	Brazil	7	6	146	2011

*Xu, Z.H. (China) – Co-shared the same position with asterisked author in the pecking order.
